# Effects of Electrical Stimulation on Peripheral Nerve Regeneration in a Silicone Rubber Conduit in Taxol-Treated Rats

**DOI:** 10.3390/ma13051063

**Published:** 2020-02-27

**Authors:** Chien-Fu Liao, Shih-Tien Hsu, Chung-Chia Chen, Chun-Hsu Yao, Jia-Horng Lin, Yung-Hsiang Chen, Yueh-Sheng Chen

**Affiliations:** 1 Department of Biological Science and Technology, School of Medicine, China Medical University, Taichung 40402, Taiwan; lcfa1974@gmail.com (C.-F.L.); chyao@mail.cmu.edu.tw (C.-H.Y.); 2 Lab of Biomaterials, Graduate Institute of Biomedical Sciences, China Medical University, Taichung 40402, Taiwan; hsuvghtc@gmail.com; 3 Department of Obstetrics and Gynecology, Taichung Veterans General Hospital, Taichung 40705, Taiwan; 4 Linsen Chinese Medicine and Kunming Branch, Taipei City Hospital, Taipei 10341, Taiwan; dal06@tpech.gov.tw; 5 Department of Bioinformatics and Medical Engineering, Department of Psychology, College of Medical and Health Science, Asia University, Taichung 41354, Taiwan; 6 Biomaterials Translational Research Center, China Medical University Hospital, Taichung 40447, Taiwan; 7 Department of Fiber and Composite Materials, Feng Chia University, Taichung 40724, Taiwan; jhlin@fcu.edu.tw; 8 Graduate Institute of Integrated Medicine, Research Center for Chinese Medicine & Acupuncture, China Medical University, Taichung 40402, Taiwan

**Keywords:** silicone rubber, electrical stimulation, peripheral nerve regeneration, taxol, macrophage

## Abstract

Taxol, a type of antimitotic agent, could modulate local inflammatory conditions in peripheral nerves, which may impair their regeneration and recovery when injured. This study provided in vivo trials of silicone rubber chambers to bridge a long 10 mm sciatic nerve defect in taxol-treated rats. It was aimed to determine the effects of electrical stimulation at various frequencies on regeneration of the sciatic nerves in the bridging conduits. Taxol-treated rats were divided into four groups (n = 10/group): sham control (no current delivered from the stimulator); and electrical stimulation (3 times/week for 3 weeks at 2, 20, and 200 Hz with 1 mA current intensity). Neuronal electrophysiology, animal behavior, neuronal connectivity, macrophage infiltration, calcitonin gene-related peptide (CGRP) expression levels, and morphological observations were evaluated. At the end of 4 weeks, animals in the low- (2 Hz) and medium-frequency (20 Hz) groups had dramatic higher rates of successful regeneration (90% and 80%) across the wide gap as compared to the groups of sham and high-frequency (200 Hz) (60% and 50%). In addition, the 2 Hz group had significantly larger amplitudes and evoked muscle action potentials compared to the sham and the 200 Hz group, respectively (*P* < 0.05). Heat, cold plate licking latencies, motor coordination, and neuronal connectivity were unaffected by the electrical stimulation. Macrophage density, CGRP expression level, and axon number were all significantly increased in the 20 Hz group compared to the sham group (*P* < 0.05). This study suggested that low- (2 Hz) to medium-frequency (20 Hz) electrical stimulation could ameliorate local inflammatory conditions to augment recovery of regenerating nerves by accelerating their regrowth and improving electrophysiological function in taxol-treated peripheral nerve injury repaired with the silicone rubber conduit.

## 1. Introduction

Chemotherapy induces peripheral neuropathy [1,2]. Taxol (paclitaxel) is a common microtubule-binding antineoplastic drug used to treat solid tumors. It binds to and stabilizes the length of microtubules, which results in microtubule dynamics suppression, leading to mitotic arrest and dividing cancer cell apoptosis [3]. In addition, exposure to taxol may cause neurodegeneration, resulting in serious side effects including myelosuppression and peripheral neurotoxicity [4]. Taxol could also induce clinical sensory neuropathy with symptoms of tingling, numbness, or burning pain. Although those symptoms in most patients could resolve within months after treatment, sensory pain may occasionally become a chronic problem [5].

It has been reported in the literature that taxol is also a potent microtubule-stabilizing drug, which can promote regeneration of injured adult central nervous system (CNS) axons [6,7]. Compared to the axons in the CNS, those in the peripheral nervous system regenerate better since their microtubules in the damaged region of the axon become less acetylated [8]. Taxol treatment has been found to notably increases microtubule acetylation [9]. My group found that excessive taxol injected in rats may hinder peripheral nerve regeneration by decreasing expression of immunoregulatory factors, macrophage invasion, and calcitonin gene-related peptide (CGRP) expression in the spine [10]. 

In the present study, we used silicone rubber conduits to assist in the regeneration of taxol-injured peripheral nerves. Because the properties of the silicone rubber are highly stable and nondegradable, it can provide a suitable and sustainable environment in the bridging conduit for regeneration of injured nerves [11,12]. In the literature, it has been reported that a weak electric field may enhance neurite outgrowth both in vitro [13,14] and in vivo [15,16]. Additionally, electrical stimulation could improve circulation to accelerate regenerative processes and ameliorate nerve functions [17]. Our group has also successfully demonstrated the beneficial effects of percutaneous electrical stimulation on long sciatic nerve defects in normal as well as diabetic rats [17,18,19,20]. However, the opposite results of electric fields on regenerating nerves have been reported, too [21,22]. Discrepant results across studies are likely due to the different frequencies of electrical stimulation used [23,24]. Furthermore, most studies have investigated the effects of electrical stimulation on nerve regeneration using only a short nerve gap less than 10 mm wide.

So far, there is still no information available in the literature addressing the effect of electrical stimulation on regeneration of dissected taxol-treated peripheral nerves repaired with a bridging conduit. Therefore, we further designed this experiment to assess whether electrical stimulation at various frequencies can assist growth of peripheral nerves in taxol-treated rats across a 10-mm gap in silicone rubber conduits. After a recovery period, the overall effects of the electrical stimulation on growing nerves were then confirmed by electrophysiological examinations, animal behavior patterns including thermal hyperalgesia, and motor coordination tests, as well as morphological observations of CGRP expression in the spine, retrograde fluorogold-labelling dorsal root ganglions (DRGs), and macrophage infiltration in regenerated nerves. These experiments were aimed to elucidate mechanisms underlying the observed effects of electrical stimulation on taxol-treated neuronal regeneration.

## 2. Materials and Methods

### 2.1. Ethical Statement

The study was conducted in accordance with the use of Laboratory Animals (National Academy Press). All protocols were approved by the Ethics Committee of the China Medical University, Taiwan (Project identification code: CMUIACUC-2018-242).

### 2.2. Experimental Design and Surgical Protocols

**Figure 1:**
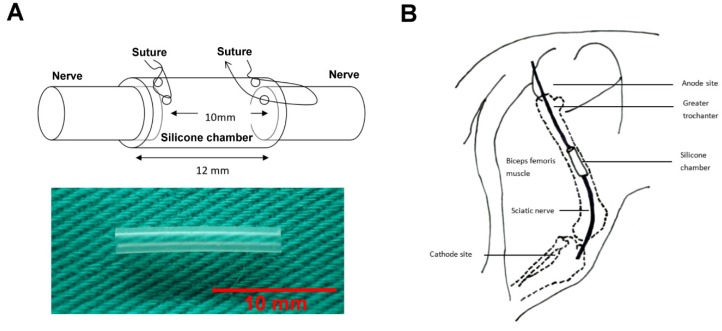
The diagrams to scale of (**A**) the implanted material in the animal and (**B**) electrode positions. Scale bar = 10 mm.

Right sciatic nerves of anesthetized female Sprague-Dawley rats were severed into proximal and distal segments. Both of the stumps were fixed in a silicone rubber chamber (Helix Medical, Inc., Carpinteria, CA, USA) with a 10-mm gap apart [25,26]. The inner diameter of the tube is 1.47 mm; outer diameter is 1.96 mm; and length is 12 mm. The diagrams to scale of the implanted material was shown in Figure 1A. Taxol (6 mg/kg) dissolved in Cremophor EL solution (Sigma Chemicals, St. Louis, MO, USA) was then injected intraperitoneally (i.p.) in the animals on days 0, 2, 4, and 6 [27]. The taxol-administered animals were divided into four groups (10 for each group): the sham controls (no electrical stimulation); groups received electrical stimulation (3 times/week for 3 weeks) at 2, 20, and 200 Hz, respectively, at the current intensity of 1 mA. The form of the electrical stimulation was constant square wave.

### 2.3. Electrical Stimulation

One week after nerve injury, a needle electrode (stainless steel, 0.35 mm OD, 12 mm length) connected to the cathode of a stimulator (Trio 300; Ito, Tokyo, Japan) was inserted aseptically into the lateral aspect of the knee and the anode at the hip joint [19,28]. The diagrams to scale of the implanted material in the animal and electrode positions is shown in Figure 1B.

### 2.4. Thermal Hyperalgesia

A hot/cold plate (Panlab, Harvard Apparatus, Holliston, MA, USA) was used to measure both hot/cold-induced pains [29]. Animal behavior was recorded for five minutes and licking latency measured [29,30,31]. The source of radiant heat (40 °C) was beneath a glass floor at the hind paw plantar region. Similarly, the rats were placed onto a cold plate apparatus at 4 °C.

### 2.5. Motor Coordination Test

Motor coordination was evaluated using Rotamex Columbus instruments (Rotamex rotarod, Columbus Instruments, Columbus, OH, USA). Accelerating rota-rod testing was performed with initial setting at 6 rpm and accelerated by 2.5 rpm every 10 s [32]. 

### 2.6. Electrophysiological Techniques

After the behavior test, electrophysiological examinations were performed, including nerve conduction velocity (NCV), amplitude, latency, and evoked muscle action potentials (MAPs) of the gastrocnemius muscles using BIOPAC Systems, Inc. (Goleta, CA, USA) [10].

### 2.7. Fluorogold Retrograde Labelling

Fluorogold (Fluorochrome, Denver, CO, USA) solution was injected using Hamilton micro-syringe into the common peroneal and posterior tibial nerves. Five days later, the animals were perfused transcardially with saline and paraformaldehyde and the L4 and L5 DRGs ipsilateral to the injury were removed. Frozen sections of the spinal cord and DRGs were then examined using a fluorescence microscope (Olympus ckx41, Center Valley, PA, USA).

### 2.8. Histological Analyses and Image Assay

The regenerated nerves were retrieved after 4 weeks of recovery from the silicone rubber conduits and the middle regions of the nerves were cut and placed in a 10% formalin solution for 24 hours of fixation. Then, 70-nm ultra-thin nerve sections were prepared and examined by transmission electron microscopy (TEM) at 100 kV (Leica, Wetzlar, Germany). The L4 spinal cord was removed, post-fixed, and treated serially with anti-CGRP antibody 1:1000 (Calbiochem, San Diego, CA, USA) and secondary antibody (Novolink Polymer RE7112). Macrophages (CD68^+^) in the distal regions of the regenerated nerves were studied in immunostaining images under an SP2/SP8X microscope (Leica, Wetzlar, Germany). An image analyzer system (Image-Pro Lite, Media Cybernetics, Rockville, MD, USA) was used to measure the ratio of positive CGRP-immunoreactive areas in the dorsal horn ipsilateral to the injury and the number of neural components [20]. 

### 2.9. Statistical Analyses

The statistical analyses of the continuous variables, category variables, and groups comparison of proportion were using Chi-square analysis of variance from SAS Enterprise Guide 7.1/JMP 14 pro (SAS Institute, Inc., Cary, NC, USA) at significance level of *P* < 0.05.

## 3. Results

### 3.1. Regeneration Across Gaps

**Figure 2:**
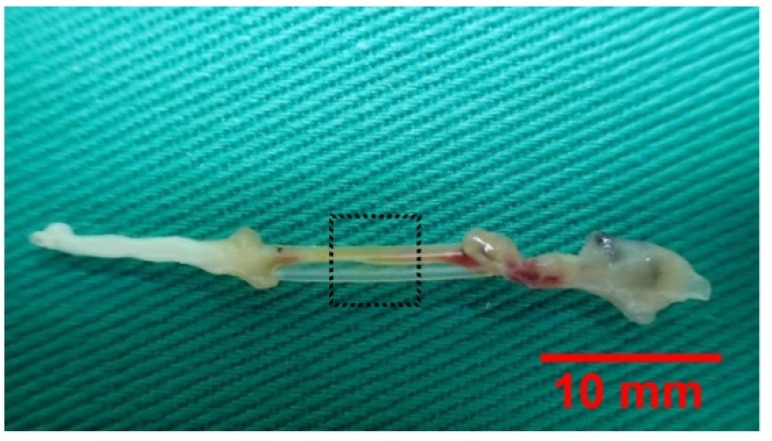
The nerve cable in the silicone rubber conduit after removal from the rat. The boxed area is where the specimens were taken for histological analysis. Scale bar = 10 mm.

After 4-week implantation, the silicone rubber conduits with the regenerated cables inside were studied. Only a thin fibrous tissue encapsulation was seen covering the bridging implants. After removing these tissues, the inner regenerated cable could be seen through the transparent tube wall (Figure 2). The overall success rate of the regenerated cable connecting the two severed nerve stumps in the sham control (no current delivered by stimulator), low- (2 Hz), mid- (20 Hz), and high-frequency (200 Hz) groups, was 60%, 90%, 80%, and 50%, respectively. The data demonstrated that cable formation within the bridging conduits was most significantly increased in the taxol-treated animals receiving low- and mid-frequency electrical stimulation.

### 3.2. Electrophysiological Measurements

**Figure 3:**
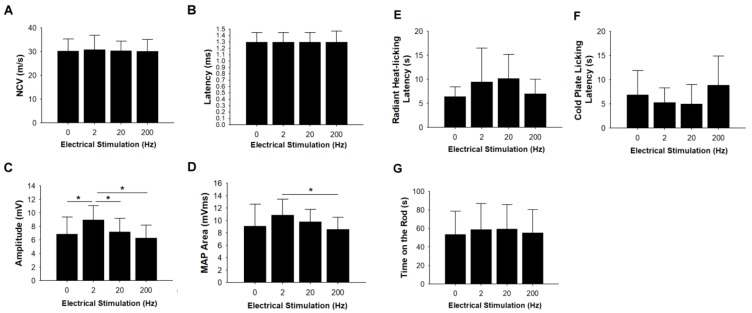
Analysis of evoked muscle action potentials (MAPs), including (**A**) nerve conduction velocity (NCV), (**B**) latency, (**C**) peak amplitude, and (**D**) area under the MAP curves. Analysis of electrical stimulation on thermal and motor coordination tests, including (**E**) radiant heat, (**F**) cold plate licking latency, and (**G**) time-on-the rod tests. * Significant differences between conditions, *P* < 0.05.

In the electrophysiological study, evident nerve conduction proved that regenerated nerves had successfully re-innervated the gastrocnemius muscle. Quantitative data (Figure 3A–D) showed that taxol-impaired nerve functions, including nerve conduction velocity and latency, were not influenced by electrical stimulation. However, amplitude of the regenerated nerves was significantly increased in the low-frequency (2 Hz) electrical stimulation group compared with the sham control, 20 Hz, and 200 Hz group; MAP area of the regenerated nerves was also significantly increased in the low-frequency (2 Hz) electrical stimulation group compared with the 200 Hz group.

### 3.3. Thermal Hyperalgesia and Motor Coordination Tests

For the thermal hyperalgesia and motor coordination tests, there were no significant differences among the groups on the radiant heat, cold plate, and motor coordination (time on the rod) (Figure 3E–G).

**Figure 4:**
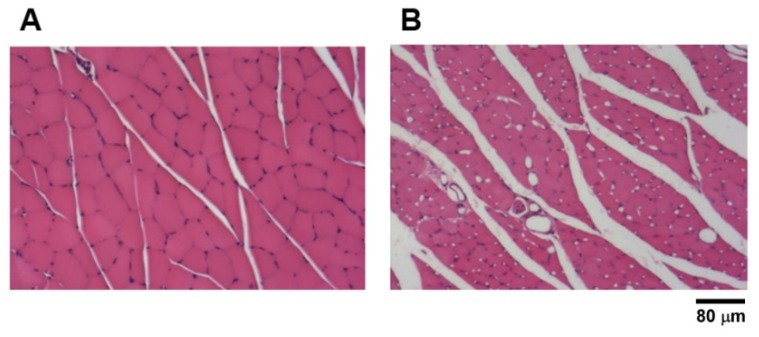
(**A**) Normal rat gastrocnemius muscle. (**B**) Evident muscle atrophy is noted in all of the taxol-treated rats with or without electrical stimulation. Scale bar = 80 μm.

Inspection through an optical microscope, morphological changes of gastrocnemius muscle are shown in Figure 4. Compared with normal muscle fibers, those from taxol-treated rats with or without electrical stimulation all showed a very severe atrophy with smaller muscle fibers and evident fatty infiltration. 

### 3.4. Fluorogold Retrograde Labelling

**Figure 5:**
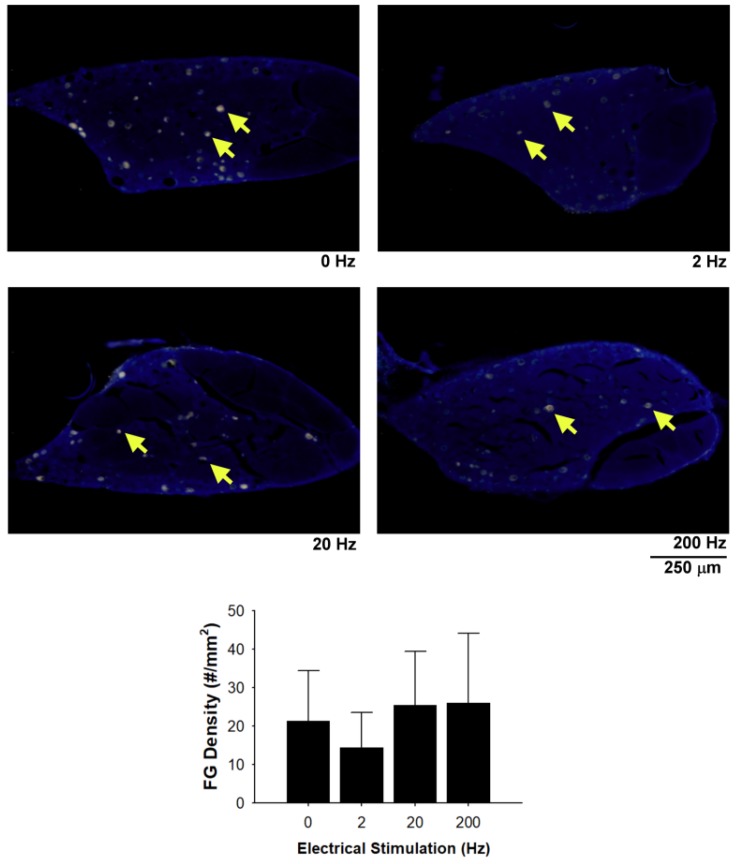
Representative images of the retrograde axonal tracing with fluorogold (arrows) in taxol-treated rats. No significant difference was seen in the number of fluorogold-labelled cell in dorsal root ganglions (DRGs) among the four groups with different frequencies of electrical stimulation. Scale bar = 250 μm.

Fluorogold-labelled cells in cryostat sections implied that regenerated axons had grown across the nerve gap and the fluorescent micrographs showed the fluorogold retrograde tracing in the DRGs, indicating successful neuronal connectivity. There were no statistically significant differences in the density of fluorogold-labelling in the DRG among the four groups (Figure 5).

### 3.5. Ultrastructural Analysis and Maturation of Regenerated Nerves

**Figure 6:**
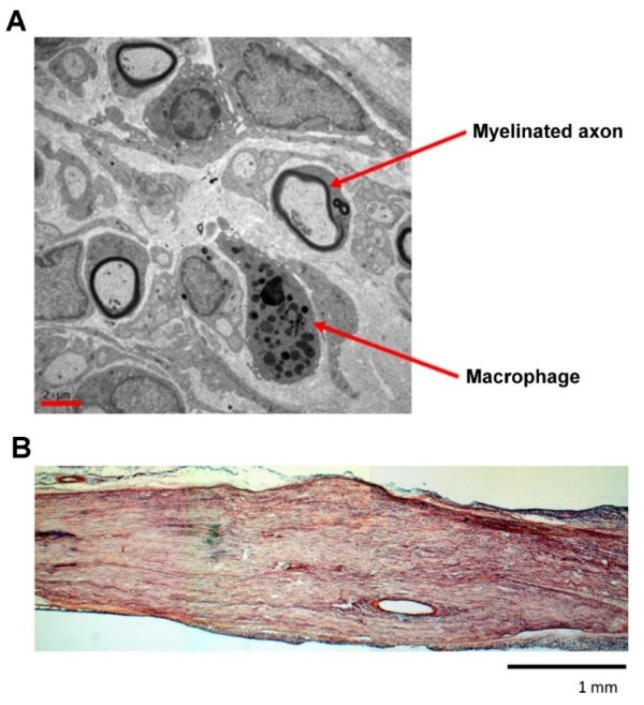
(**A**) A representative transmission electron microscopy (TEM) image showing the nerve in taxol-treated rats. Scale bar = 2 μm. (**B**) A longitudinal view of regenerated nerves in taxol-treated rats. Scale bar = 1 mm.

TEM showed that endoneurial macrophages were very close to Schwann cells wrapping the myelinated axon (Figure 6A). Figure 6B shows a representative longitudinal view of regenerated nerves with a bundle of newly-developed nerve fibers. 

**Figure 7:**
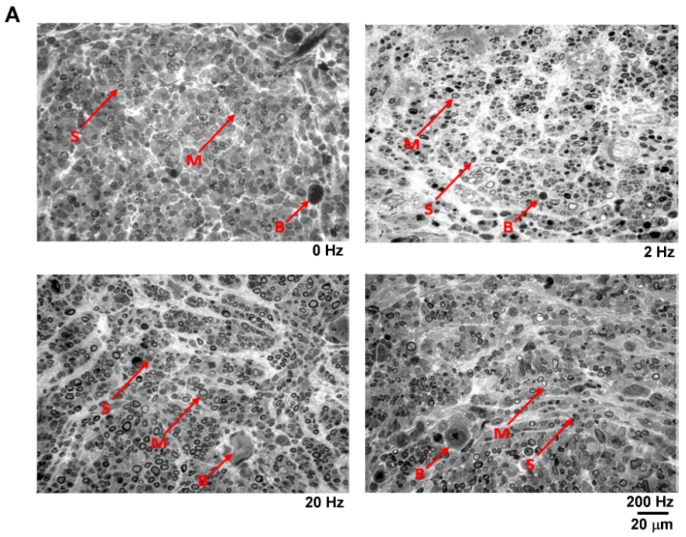

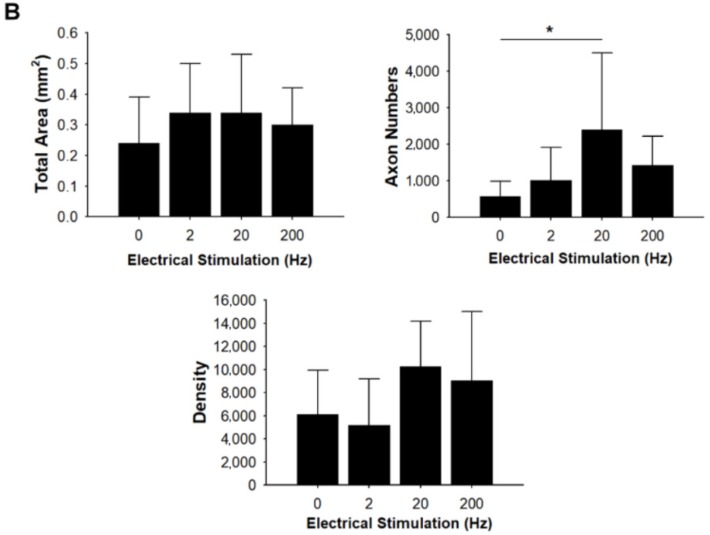
Effects of electrical stimulation on axon regeneration in taxol-treated rats. (**A**) Representative micrographs of nerve tissues including myelinated axon (M), blood vessel (B), and Schwann cell (S). (**B**) Morphometric comparisons of regenerated nerves. * Significant differences between conditions, *P* < 0.05. Scale bar = 20 μm.

Using the light microscopy, the nerves in the control group exhibited an immature structure with numerous Schwann cells accompanied with dispersed myelinated axons and blood vessels. In all electrical stimulation groups, the successfully regenerated nerves exhibited a relatively mature structure with more myelinated axons. Morphometric data revealed that the axon number was significantly increased in the mid-frequency (20 Hz) electrical stimulation group compared with the control group. However, no statistically significant differences were found in total area and axon density in the successfully regenerated nerve cables among groups (Figure 7). 

### 3.6. Recruited Macrophages in the Distal Nerve Ends

**Figure 8:**
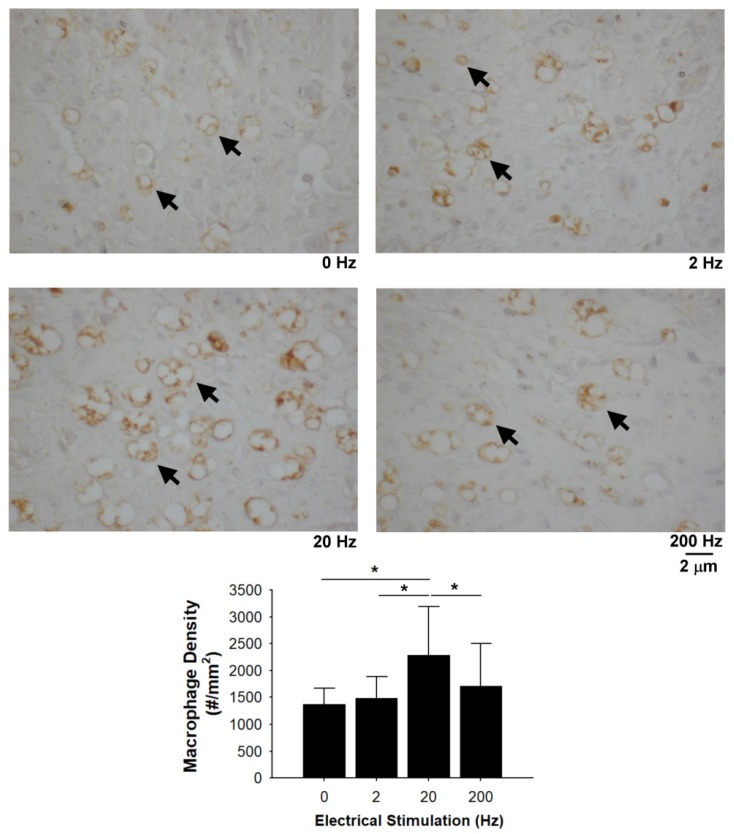
Effects of electrical stimulation on macrophage infiltration in taxol-treated rats. Representative images of the macrophages (arrows) and quantitation of macrophage infiltration. * Significant differences between conditions, *P* < 0.05. Scale bar = 2 μm.

Figure 8 depicts the expression of macrophage CD68 in regenerated nerves. Quantitative immunostaining data for CD68 reflected that mid-frequency (20 Hz) electrical stimulation could significantly increase macrophage infiltration into the injured sites compared with the sham control, 2 Hz, and 200 Hz groups. 

### 3.7. CGRP Immunoreactivity in the Dorsal Horn

**Figure 9:**
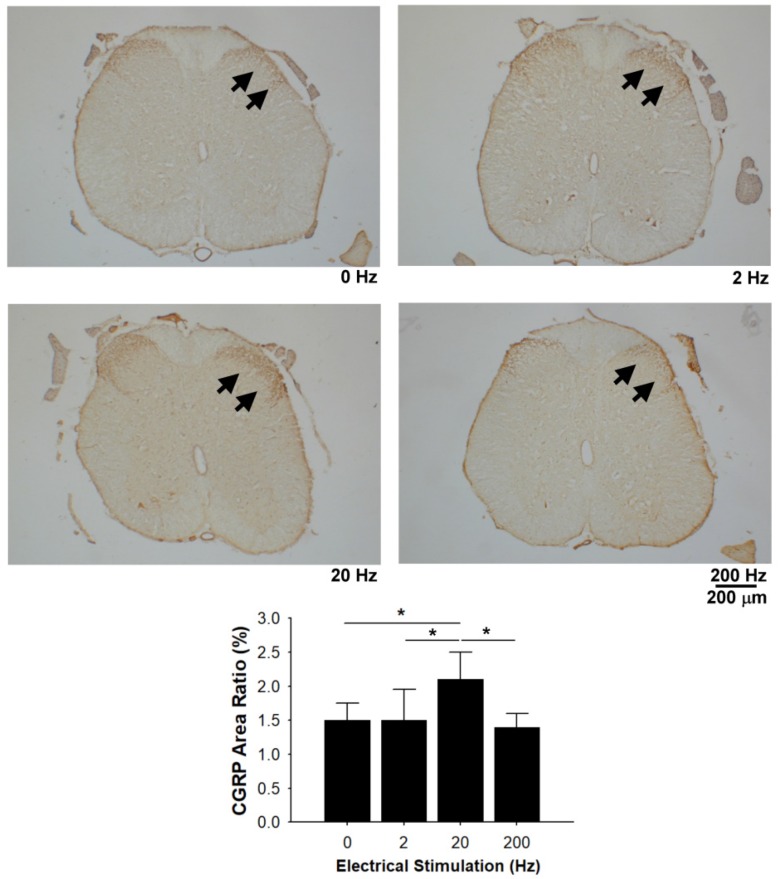
Effects of electrical stimulation on calcitonin gene-related peptide (CGRP) expression in taxol-treated rats. Representative images of CGRP-labelled fibers (arrows) and comparison for the ratio of CGRP expression area. * Significant differences between conditions, *P* < 0.05. Scale bar = 200 μm.

Immunohistochemical staining demonstrated the presence of CGRP-labelled fibers in the area of lamina of the dorsal horn in all of the rats. Quantitative data revealed that CGRP expression was significantly increased in the mid-frequency (20 Hz) stimulation group compared with the sham control, 2 Hz, and 200 Hz groups (Figure 9).

## 4. Discussion

Taxol is an antineoplastic agent that promotes microtubule assembly which could lead to peripheral neuropathy. A variety of neuroprotective drugs have been developed to reduce the occurrence of neurotoxicity due to taxol [33]. Our previous study demonstrated that taxol could cause worsening of local inflammation and hinder peripheral nerve regeneration [10]. In our other study, we found that regenerated nerves in normal rats receiving electrical treatment, especially at 2 Hz, had a more mature structure with increased myelinated fibers and blood vessels compared with the controls receiving no electrical stimulation [28]. In the present study, we further assessed the influence of electrical stimulation at different frequencies on regenerating sciatic nerves across a large defect repaired using a silicone rubber conduit in a taxol-treated rat model. 

Previous studies have studied the effects of electrical stimulation on peripheral nerve regeneration in animals. It has been reported that electrical stimulation could accelerate recovery from facial paralysis after a crush injury in rats [34]. It has also been shown that high-voltage (intensity) electrical stimulation can improve functional recovery with more matured regenerated axons by suppressing macrophage levels after sciatic nerve crush in animals [35,36]. In these nerve crush studies, the basal lamina in the nerve trunks could still be intact. It is absolutely impossible to exist in a nerve transection model [37]. Some other studies have demonstrated that electrical stimulation may accelerate peripheral nerve regeneration through expression of nerve injury/regeneration-associated genes [38,39]. Variables include the types of electrical stimulation (constant/pulse of direct/alternating current), stimulation parameters (frequency and intensity), and the sites of placement of electrodes have also been studied [23]. In the research of nerve conduits, numerous materials have been developed to repair peripheral nerve injury, such as silicone rubber [23,40], gelatin [41], polyglycolic acid [42], polyurethane [41], poly(l-lactide-co-caprolactone) [43], and so on. These nerve conduits are usually designed as a tubular structure, which could provide mechanical orientation and confinement to aid growing nerve fibers. In this study, nondegradable silicone rubber was used to make the bridging conduit for its stable properties, which could provide a continuous support for the regenerating nerves. Thus, some unexpected factors, such as the situation that affects nerve growth after material disintegration, can be ruled out. However, much clinical evidence reports issues including pain, nerve compression, tension at the suture site, and the need of a second surgery for removal after regeneration has occurred [44,45,46].

In the present study, histomorphometric results demonstrated that electrical stimulation at a low- (2 Hz) to mid-frequency (20 Hz) may increase the regenerated nerve maturation that successfully crosses the gap in the taxol-treated rats. In addition, regenerating nerves stimulated at 2 Hz exhibited a significantly higher amplitude and MAP area. The mechanism in which frequency ameliorates neuronal regeneration is still unclear. However, it has been found that the electrical stimulation could cause muscle contractions to accelerate blood flow in regenerated nerves [47], which may be caused by the reflex arc [48]. As we know, providing enough blood is critical for the success of nerve regeneration. Therefore, it is conceivable that applying external electrical stimulation in taxol-treated rats could increase their peripheral perfusion to provide more nutrients for regenerating nerves, thus acquiring a higher chance of successful nerve regeneration. In addition, we found that electrical stimulation at mid-frequency (20 Hz) enhanced CGRP expression in the dorsal horn. Since CGRP is a nerve regeneration-promoting peptide [48], the increased CGRP expression was also beneficial to the regenerating nerves. Third, we found that electrical stimulation at mid-frequency could promote the macrophage infiltration into the endoneurium following nerve injury in taxol-treated rats. It has been reported in the literature that the macrophages not only can remove myelin debris from the degenerative process, but also secrete nerve-growth factors [49,50]. This could be another reason that electrical stimulation treatment could lead to the nerve regenerative response enhancement. By comparison, it was found that high-frequency electrical stimulation at 200 Hz used in the present study could not enhance formation of a nerve fiber cable across the nerve gap. One possible explanation is that the taxol-treated rats in the high-frequency electrical stimulation group had relatively fewer macrophages in the distal sciatic nerve, delaying Wallerian degeneration with less secretion of nerve-growth factors. Similar results have been reported; that high-frequency electrical stimulation could induce early axonal degeneration and neural injury, slowing the rate of axon growth and remyelination [24,28]. However, electrical stimulation also appears to exert nerve growth-promoting effects as shown in the abovementioned results. Thus, it is conceivable that electrical stimulation may possibly have a dual effect on regenerating nerves, depending on its stimulation parameters.

There were limitations to this study that should be addressed. It is known that significant species variability exists in nerve regeneration processes and, as such, caution must be exercised when attempting to extrapolate the results of animal studies. Moreover, single methods of peripheral nerve regeneration measurement yield only limited data; therefore, a better understanding of peripheral nerve regeneration is possible by combining more evaluation methods, such as immunohistochemical observations, electrophysiological analyses, and morphometric comparisons.

## 5. Conclusions

To the best of our knowledge, this is the first study to investigate the effects of electrical stimulation at different frequencies on regeneration of taxol-treated rat peripheral nerve in silicone rubber conduits. Results of this study may provide a basis for considering electrical stimulation at low- to mid-frequencies as a complementary treatment in patients who experience taxol-related neuropathy.
